# Unraveling Early Signs of Navigational Impairment in APPswe/PS1dE9 Mice Using Morris Water Maze

**DOI:** 10.3389/fnins.2020.568200

**Published:** 2020-12-15

**Authors:** Smitha Karunakaran

**Affiliations:** Centre for Brain Research, Indian Institute of Science, Bangalore, India

**Keywords:** Alzheimer's disease, search strategy, morris water maze, APPswe/PS1dE9, mild behavioral impairment, circling strategy

## Abstract

Mild behavioral deficits, which are part of normal aging, can be early indicators of an impending Alzheimer's disease. Using the APPswe/PS1dE9 (APP/PS1) mouse model of Alzheimer's disease, we utilized the Morris water maze spatial learning paradigm to systematically evaluate mild behavioral deficits that occur during the early stages of disease pathogenesis. Conventional behavioral analysis using this model indicates that spatial memory is intact at 2 months of age. In this study, we used an alternative method to analyze the behavior of mice, aiming to gain a better understanding of the nature of cognitive deficits by focusing on the unsuccessful trials during water maze learning rather than on the successful ones. APP/PS1 mice displayed a higher number of unsuccessful trials during the initial days of training, unlike their wild-type counterparts. However, with repeated trial and error, learning in APP/PS1 reached levels comparable to that of the wild-type mice during the later days of training. Individual APP/PS1 mice preferred a non-cognitive search strategy called circling, which led to abrupt learning transitions and an increased number of unsuccessful trials. These findings indicate the significance of subtle intermediate readouts as early indicators of conditions such as Alzheimer's disease.

## Introduction

Behavioral impairments lacking prominent clinical symptoms can be indicative of normal aging or mild cognitive impairment. However, in some cases, these impairments develop into Alzheimer's disease (AD), a progressive neurodegenerative disorder that is neuropathologically characterized by extracellular senile plaques, formed by amyloid-β (Aβ) accumulation and intracellular neurofibrillary tangles, composed of a hyperphosphorylated microtubule-associated protein called tau (Ashe and Zahs, 2010; Tarawneh and Holtzman, 2012). Carriers of mutations associated with familial autosomal-dominant AD are known to develop subtle cognitive deficits <25 years before they develop dementia (Mondadori et al., 2006; Mosconi et al., 2006). Therefore, timely and accurate diagnosis is critical for the development of treatments for the initial stages of AD. Biomarkers directly or indirectly relevant to the histopathology of AD, such as blood and cerebrospinal fluid biomarkers, along with PET ligands are highly valuable in this scenario (Dubois et al., 2014). Recently, early behavioral markers that recognize and predict underlying pathology have gained importance in the diagnosis of AD (Webster et al., 2014; Hassenstab et al., 2016). For example, significant episodic memory impairments are reported 10–12 years before symptom onset in familial AD patients (Bateman et al., 2012). Thus, subtle behavioral markers can make early diagnosis more feasible, opening up a new avenue in AD prevention and therapeutics. Therefore, the aim of this study was to identify early behavioral markers in a well-studied mouse model of AD amyloidosis, APPswe/PS1dE9 (APP/PS1: Jankowsky et al., 2001; Borchelt Line 85).

Transgenic mouse models designed based on the genes underlying AD have increased our knowledge of AD mechanisms tremendously (LaFerla and Green, 2012; Webster et al., 2014). Although no single mouse model fully exemplifies human AD pathology and cognitive deficits (Jankowsky and Zheng, 2017), transgenic mice with an established underlying neuropathology are excellent model systems to study specific questions, such as early behavioral impairments. Identifiable amyloid deposition in this APP/PS1 model has been observed at approximately 4–6 months of age, but no identifiable plaques have been observed at 2 months (Jankowsky et al., 2004; Garcia-Alloza et al., 2006). This provides us with a window to study the earliest signs of mild behavioral impairments that are independent of Aβ plaque deposition.

Cognitive deficits in this model were first described at 3 to 4 months of age in the Radial Arm Water Maze spatial working memory task (Jankowsky et al., 2001; Park et al., 2006). Other studies reported normal performance at 7 months of age and reduced performance at 13 months (Volianskis et al., 2010) or at 10–15 months (Sood et al., 2007) with the same task. However, Morris water maze (MWM) deficits are reported at just 6 months of age in APP/PS1 (Cao et al., 2007; Ding et al., 2008) and are associated with increased amyloid deposition (Cao et al., 2007; Reiserer et al., 2007; Ding et al., 2008). Deficits have also been well characterized across the lifespan of other related amyloid lines in water-based spatial working memory tasks (Gong et al., 2004; Trinchese et al., 2004; Lalonde et al., 2005; Puzzo et al., 2009; Cramer et al., 2012).

All MWM experiments performed with APP/PS1 mice thus far have used conventional behavioral readouts based on average performances, such as escape latencies and probe tests, to arrive at a conclusion (**Table 2**). These readouts do not effectively identify subtle behavioral deficits in APP/PS1 with high sensitivity and an acceptable degree of specificity due to their focus on endpoints. Therefore, this study introduces a modified method of analysis by focusing more on the individual learning sequences of mice. Our study revealed cognitive deficits in 2-month-old male APP/PS1 in comparison to their age-matched WT counterparts. Water maze learning forces mice to develop efficient navigational strategies that focus on local associations to find the hidden platform. However, the specific mechanisms of strategic searching toward the goal remain unclear (Sutton and Barto, 1998; Botvinick et al., 2009). Detailed search strategy recruitment-based studies have been conducted in many AD mouse models, such as TgCRND8 mice (Janus, 2004; Granger et al., 2016), PDAPP mice (Brody and Holtzman, 2006), TgF344 mice (Berkowitz et al., 2018), tetO-APPswe/ind 102 line (Chiang et al., 2018), and APP21 rats (Weishaupt et al., 2018). However, only two studies (Schrott et al., 2015; Zhang et al., 2016) have briefly attempted search strategy classification in APP/PS1 mice. This prompted a further analysis of our data based on the hierarchical recruitment of strategies during MWM learning. Our detailed longitudinal analysis revealed statistically significant differences in the pattern of search strategies employed by 2-month-old male APP/PS1 and age-matched WT mice. Taken together, our study investigates mild behavioral deficits in MWM learning during an early stage of disease pathogenesis in an amyloidogenic mouse model of AD.

## Materials and Methods

### Experimental Animals

The generation, care, and use of mice, as well as all experimental procedures, were approved by the Institutional Animal Ethics Committee of the Indian Institute of Science, Bangalore. These animal experiments also complied with the Animal Research: Reporting of *In Vivo* Experiments (ARRIVE) guidelines. Transgenic mice B6C3-Tg (APPswe/PS1dE9) 85Dbo/J (https://www.jax.org/strain/005864) obtained from The Jackson Laboratory were kindly provided by Prof. Vijayalakshmi Ravindranath, Director, Center for Brain Research, Bangalore, India. Wild-type (WT) and APP/PS1 mice were bred at the Institutional Central Animal Facility, were housed in standard mouse cages under conventional laboratory conditions (12-h dark and 12-h light cycle, constant temperature and humidity), and were given food and water *ad libitum*. We performed behavioral experiments using male and female APP/PS1 and WT mice, with WT mice serving as the control.

No statistical methods were used to predetermine sample sizes; our sample sizes are instead similar to those generally employed in the field. The sample size per group is mentioned in the respective figure legends, and no samples were excluded from any of the experiments described herein, unless otherwise mentioned in the analysis. The WT and APP/PS1 mice were assigned randomly to respective groups based on their genotype. Different litters of the same age group were taken and were divided into control and experimental groups. The mice were housed individually for 3 days and were handled for 5 min every day prior to behavioral testing. All behavioral experiments were conducted at approximately the same time during the light cycle (9:00–15:00) by the same experimenter.

### Morris Water Maze

The MWM experiments were conducted, as described in previous studies (Vorhees and Williams, 2006; Ruediger et al., 2012; Karunakaran et al., 2016), on two separate days with two batches of male animals. Each batch had nine WT and nine APP/PS1 male mice. The results of two replicates (batches) of the same experiment have been combined and represented as *n* = 18. Results from individual data sets have been represented in the supplementary data. A separate set of MWM experiments was also performed with seven male WT and APP/PS1 mice each.

**Figure 1:**
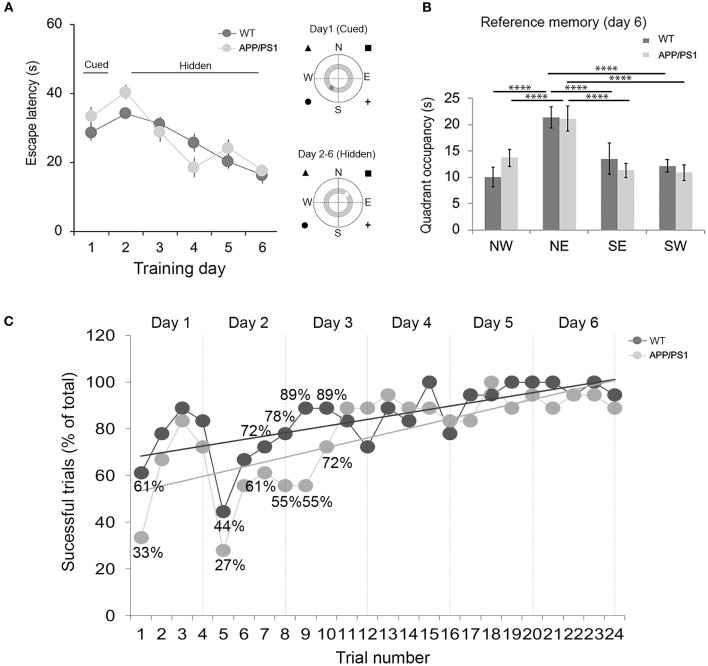
Evaluation of spatial learning in APP/PS1 mice using the Morris water maze [**(A)**, to the right] Illustration depicting the water maze pool virtually divided into four equal quadrants (NE, NW, SE, and SW) with four starting positions (N, S, E, and W), along with the extra maze cues. The inner gray circle represents the annulus zone. [**(A)**, to the left] Performance was evaluated as average escape latency between 2-month-old wild-type (WT; dark circle) and APP/PS1 (light circle) mice. **(B)** Reference memory was evaluated 2 h after the final trial on day 6. **(C)** Performance was evaluated as the percentage of successful trials between 2-month old WT and APP/PS1 mice during learning acquisition from days 1 to 6. Greater than 10% differences in successful trials across days 1, 2, and 3 between WT and APP/PS1 are highlighted with corresponding numbers. The trendline depicts the percentage difference across successful trials across WT (dark gray) and APP/PS1 (light gray). Data are presented as mean ± SEM; *n* = 18. ****P < 0.0001.

The experimental setup consisted of a large circular black pool (diameter: 180 cm) filled with water, maintained at 22–25°C and a depth of 60 cm, in a room with visual cues (triangle, square, cross, and circle) under soft and diffuse light conditions. The water was made opaque by adding non-toxic white paint (Faber Castell Tempera Fun Paint). The pool was then virtually divided into four equal quadrants (NE, NW, SE, and SW; see Figure 1A for illustration) with four starting positions (N, S, E, and W). The platform (visible or hidden) was placed in the middle of one of the virtual quadrants, and this position was maintained across the training. An overhead video camera was used to record and monitor behavior.

### Visible Platform (Cued) Testing

The escape platform (diameter: 10 cm) was positioned at a fixed location in the SW quadrant, 0.5 cm above the water surface, and was marked with a red tape. The 1st day (day 1) of training involved four trials by placing the animal in the water facing the pool wall at one of the four starting positions (N, S, E, and W). The animals were given 60 s to locate the platform and were returned to their home cage under an infrared lamp for 5 min between the trials. The animals had to remain on the platform for at least 5 s for the trial to be considered a success.

### Hidden Platform (Place) Testing

From days 2 to 6, the escape platform was positioned at a fixed location in the NE quadrant, 2.54 cm below the water surface and without the red tape. The acquisition training involved four trials, each lasting 60 s. Mice that failed to reach the platform were guided to the location by the experimenter, allowed to stay on the platform for 15 s before being removed from the water, and then returned to their home cage under an infrared lamp for 5 min between the trials. Learning acquisition with the hidden platform ended on day 6 with a single probe test for 60 s, either 2 or 24 h after the last training trial. The probe test was conducted in the absence of a platform to assess the reference memory of the animals for the learned position of the platform.

### Spatial Reversal Learning

Reversal trials were conducted from days 7 to 11 with the platform hidden and placed in the opposite quadrant (SW). Reversal learning trials ended on day 11 with a single probe test for 60 s, 2 h after the last learning trial. The probe test was conducted in the absence of a platform to assess the reference memory of the animals for the new platform location.

### Search Strategy Analysis

The path traveled by the mouse was video-tracked by an overhead camera. The video files of each trial were processed frame by frame using OpenCV. In the first frame, Hough Circle Transform was used to detect the outer edge of the maze. Canny edge and contour detection were used to identify the location of the mouse, and its coordinates were stored in a list. The path trace of the mouse was created by connecting the coordinates from the list, and the total distance traveled (in pixels) was calculated as the sum of their Euclidean distances, which was then converted to meters. Average speed was calculated from the total distance traveled divided by the total time taken.

For search strategy analysis, videos were randomized and initially scored for strategy by a single investigator, who assigned a predominant search strategy to each trial using a categorization scheme similar to those developed previously (Wolfer and Lipp, 2000; Graziano et al., 2003; Lang et al., 2003; Janus, 2004; Brody and Holtzman, 2006; Ruediger et al., 2012; Vouros et al., 2018). The strategies were further validated independently by two more investigators in the laboratory. When mice occasionally switched strategies during a trial, the strategy that best described the majority of the swim path was assigned. Conditions to delineate search strategies were defined as: thigmotaxis, >65% of the time 5–10 cm closer to the pool wall; random search, >70% coverage of the pool area; scanning, <70% random search inside the annulus; chaining, > 65% of the time along the annulus zone; directed search, >80% of the time the search was directed toward the target quadrant; focal search, >80% of the time in the target quadrant closer to the hidden platform; direct swim, 100% in the target quadrant closer to the hidden platform; and circling, when an animal performed tight repeated loops (Janus, 2004; Brody and Holtzman, 2006; Chiang et al., 2018), not wider concentric loops, anywhere in the pool. The results are expressed as percentage of incidence (% incidence) of each search strategy and search strategy habits by day of training over the total population of mice. A sequence of at least three trials with the same strategy was defined as a strategy block. Total block length is the sum of all blocks per strategy per mouse.

### Statistical Analysis

Data analysis was performed using Prism 7 (GraphPad Software Inc.). The statistical analyses were designed with the assumption of normal distribution and similar variance among groups. They were performed using two-tailed unpaired Student's *t*-test for paired comparisons and two-way repeated-measures analysis of variance (ANOVA) followed by *post hoc* tests for time × group comparisons, and two-way ANOVA followed by *post hoc* tests was performed when two factors were compared. The results are presented as mean ± SEM. The statistical design for each experiment can be found in the respective figure legend section. The results were considered significant at *p* < 0.05.

### Ethics Approval

This animal study was reviewed and approved by the Institutional Animal Ethics Committee of the Indian Institute of Science, Bangalore.

## Results

### Individual Latency Curves of APP/PS1 Mice Oscillate Substantially Early During Learning Acquisition

The mice were trained on day 1 on the cued water maze. Escape latency decreased over the four trials, both in the WT and the APP/PS1 [Figure 1A; day 1: two-way repeated measures ANOVA: training effect: *F*_(3, 51)_ = 13.78, *P* < 0.0001; genotype effect: *F*_(1, 17)_ = 1.806, *P* = 0.1966; interaction: *F*_(3, 51)_ = 3.105, *P* = 0.8177]. This indicated that the APP/PS1 mice did not differ in visual function, swimming ability, and motivation to escape from the pool. From day 2 to 6, the mice were taken for hidden platform testing (Figure 1A). Monitoring of primary latency during acquisition trials revealed a progressive and statistically significant decrease in latencies across WT and APP/PS1 mice. At the end of 6 days of training, both APP/PS1 and WT mice performed similarly [days 2–6: two-way repeated-measures ANOVA: training effect: *F*_(4, 68)_ = 26.12, *P* < 0.0001; genotype effect: *F*_(1, 17)_ = 0.0121, *P* = 0.9134; interaction: *F*_(4, 68)_ = 2.295, *P* = 0.0681]. Spatial reference memory was assessed on day 6 (Figure 1B), 2 h after the last training trial. Both APP/PS1 and WT mice exhibited a significant preference for the target quadrant [two-way ANOVA: *F*_(3, 136)_ = 44.4, *P* < 0.0001; genotype effect: *F*_(1, 136)_ = 0.0557, *P* = 0.8137; interaction: *F*_(3, 136)_ = 3.845, *P* = 0.0111]. Tukey's multiple-comparisons test further indicated statistically significant differences between the time spent in the target quadrant (NE) *vs*. the SW, SE, and NW quadrants in both WT and APP/PS1 mice. We further tested long-term reference memory at 24 h (day 7) in a separate batch of WT and APP/PS1 mice to check whether APP/PS1 mice maintain the strategies acquired during learning [Supplementary Figure 1A: two-way repeated-measures ANOVA; day 1: training effect: *F*_(3, 18)_ = 3.599, *P* = 0.0339; genotype effect: *F*_(1,6)_ = 4.219, *P* = 0.0858; interaction: *F*_(3, 18)_ = 2.311, *P* = 0.1106; days 2–6: training effect: *F*_(4, 24)_ = 4.56, *P* = 0.0070; genotype effect: *F*_(1, 6)_ = 0.8192, *P* = 0.4003; interaction: *F*_(4, 24)_ = 0.3855, *P* = 0.8168]. During the probe trial, both APP/PS and WT mice exhibited increased preference for the target quadrant [Supplementary Figure 1B: two-way ANOVA followed by Tukey's *post hoc* test: *F*_(3, 48)_ = 31.02, *P* < 0.0001; genotype effect: *F*_(1, 48)_ = 0.9173, *P* = 0.3430; interaction: *F*_(3, 48)_ = 0.3173, *P* = 0.8128]. Furthermore, there were no significant differences between WT and APP/PS1 mice with regard to average speed (Supplementary Figure 2A: two-tailed unpaired Student's *t-*test; WT *vs*. APP/PS1, *t* = 1.924, df = 214, *P* = 0.0556) and distance traveled (Supplementary Figure 2B: two-tailed unpaired Student's *t*-test; WT *vs*. APP/PS1, *t* = 0.9387, df = 214, *P* = 0.3489) during the entire course of MWM learning.

Tangible differences emerged when we evaluated the percentage of successful trials during individual days of training between WT and APP/PS1 mice (Figure 1C; Table 1). The percentage of successful trials was highly compromised in APP/PS1 compared to WT mice, particularly on days 1–3 of training, and improved later to reach values comparable to WT on days 4–6 with repeated training. For example, on day 3, the total number of successful trials during trial 1 was 16/18 (88.8%) in WT in comparison to 10/18 (55.5%) in APP/PS1; meanwhile, on day 5, the total number of successful trials during trial 1 was 17/18 (94.4%) in WT in comparison to 15/18 (83.3%) in APP/PS1. Ultimately, APP/PS1 made more errors in finding the hidden platform during the initial days of training, but with repeated trials, they performed at a level comparable to the WT mice.

**Table 1:** Percentage of successful trial analysis.

	**Total number of successful trials**				**% of successful trials**			
	**Trial 1**	**Trial 2**	**Trial 3**	**Trial 4**	**Trial 1**	**Trial 2**	**Trial 3**	**Trial 4**
**Wild type**								
Day 1	11	14	16	15	61.1	77.7	88.8	83.3
Day 2	8	12	13	14	44.4	66.6	72.2	77.7
Day 3	16	16	15	13	88.8	88.8	83.3	72.2
Day 4	16	15	18	14	88.8	83.3	100	77.7
Day 5	17	17	18	18	94.4	94.4	100	100
Day 6	18	17	18	17	100	94.4	100	94.4
**APP/PS1**								
Day 1	6	12	15	13	33.3	66.6	83.3	72.2
Day 2	5	10	11	10	27.7	55.5	61.1	55.5
Day 3	10	13	16	16	55.5	72.2	88.8	88.8
Day 4	17	16	16	15	94.4	88.8	88.8	83.3
Day 5	15	18	16	17	83.3	100	88.8	94.4
Day 6	16	17	17	16	88.8	94.4	94.4	88.8

### APP/PS1 Mice Deploy Qualitatively Different Rules and Search Strategies During Water Maze Navigation

**Figure 2:**
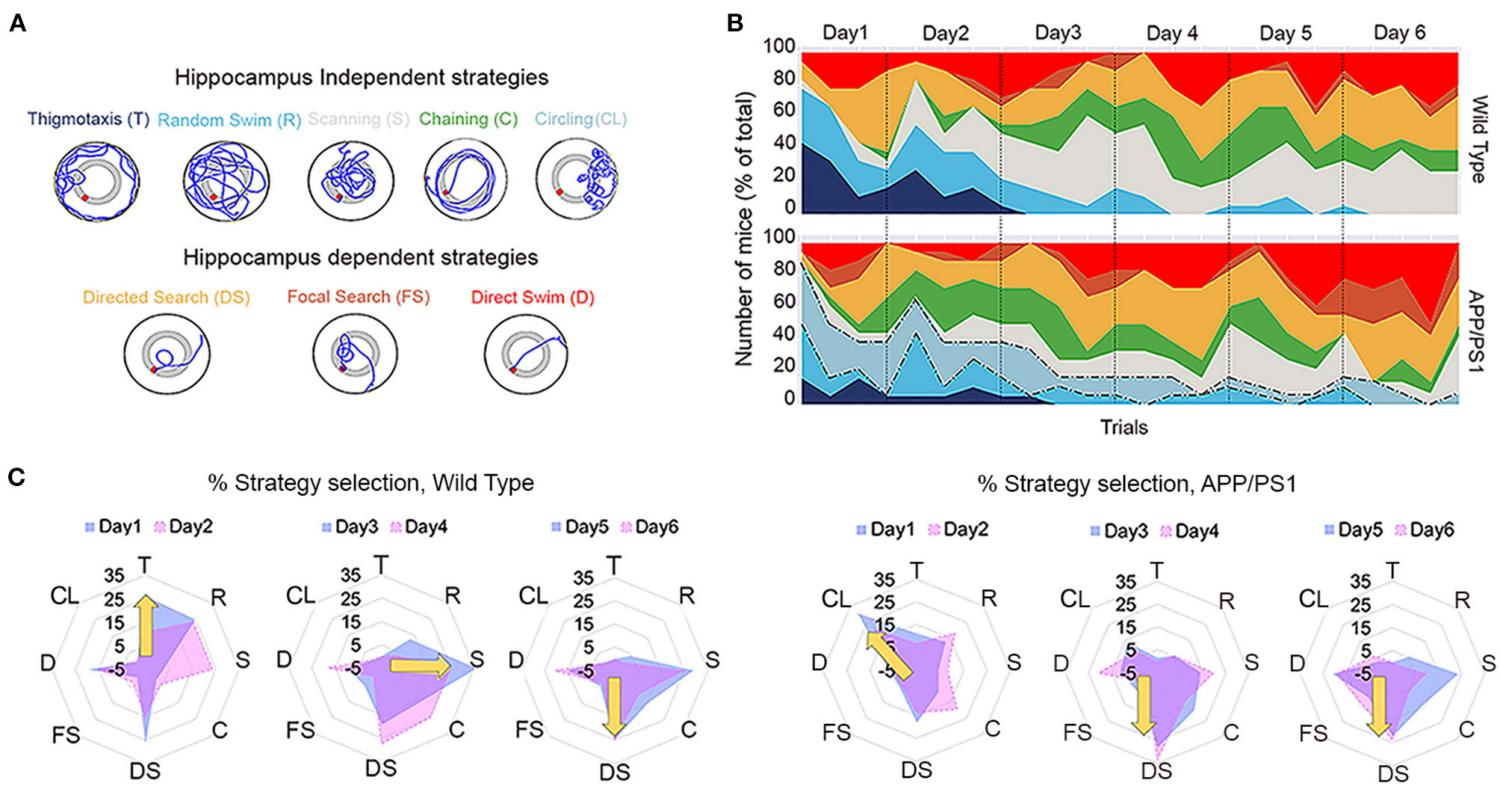
Sequential strategies deployed by wild-type (WT) and APP/PS1 mice during Morris water maze learning. **(A)** Schematic representation, color code, and abbreviations for each search strategy. The strategies have been classified as hippocampus independent (T, R, S, C, and CL) and hippocampus dependent (DS, FS, and D). **(B)** The average prevalence of each strategy by trials across days of training is shown. Note the circling strategy preference (dotted lines) of APP/PS1 during water maze learning. **(C)** Radar plots describing the day-wise average percentage strategy preference during learning by APP/PS1 (right) mice in comparison to WT (left). Note the clear shifts in strategy preference from global to local to spatial in WT (yellow arrow) and the preference of APP/PS1 (yellow arrow) toward circling, followed by an abrupt shift to spatial strategies. Data are presented as mean ± SEM; *n* = 18.

In addition to conventional readouts such as escape latency, we further augmented the strength of the analysis by classifying the behavior of individual mice based on the search strategies that they utilized to find the platform during the successive days of training. The search strategy normally evolves over time from the more non-spatial solutions used during the initial trials to the more direct spatial approaches. We cataloged all MWM studies conducted so far in APP/PS1 mice (Table 2); however, a detailed classification of search strategies has not been reported in any of these studies. It is well-known that the hippocampal integrity is essential for spatial memory, although other brain structures may also affect spatial memory by influencing spatial navigation and motor performances (D'Hooge and De Deyn, 2001). Therefore, we broadly classified the search patterns into two categories: (1) hippocampus-independent strategies, frequently used by mice that did not find the platform within the allotted time, which is highly non-spatial in nature, or (2) hippocampus-dependent spatial strategies, used by mice that learned the trained location and swam directly to it (Figure 2A).

**Table 2:** Summary of analysis done on Morris water maze data across different labs with APP/PS1 mice.

**Lab/year**	**Gender**	**Age**	**Analysis**
Gong et al. (2019)	Female	7 months	Escape latency, probe test, average speed
Li et al. (2019)	Male	8 months	Escape latency, probe test, speed, path length
Vartak et al. (2019)	Not mentioned	18 months	Escape latency, probe test, speed, distance traveled, platform crosses
Zhang et al. (2016)	Male	8 months	Escape latency, probe test, strategy
Wang et al. (2016)	Male	6 months	Escape latency, probe test
Schrott et al. (2015)	Not mentioned	8 months	Escape latency, probe test, speed, distance traveled, search strategy
Kim et al. (2015)	Male	10.5 months	Escape latency, probe test, velocity
Kummer et al. (2014)	Male and Female	4 months	Escape latency, distance traveled
Edwards et al. (2014)	Not mentioned	3.6, 9.3, and 14.8 months	Escape latency, probe test, mean velocity, distance traveled
Gallagher et al. (2013)	Female	9 months	Escape latency
Zhang et al. (2011, 2013)	Male	12–13 months	Escape latency, probe test
Hanson et al. (2012)	Male	11 months	Escape latency, path length
Su et al. (2012)	Male and female	7 months	Escape latency, probe test, average speed, distance traveled
Ma et al. (2012)	Male and female	10–12 months	Escape latency, probe test
Wen et al. (2011)	Male	7–8 months	Escape latency
Huang et al. (2011)	Male	7 months	Escape latency, probe test
Montgomery et al. (2011)	Female	12 months	Path length, probe test
Park et al. (2010)	Female	8 months	Escape latency, probe test
Ding et al. (2008)	Male	6 months	Escape latency, probe test
Cao et al. (2007)	Male	6 months	Escape latency, probe test

**Figure 3:**
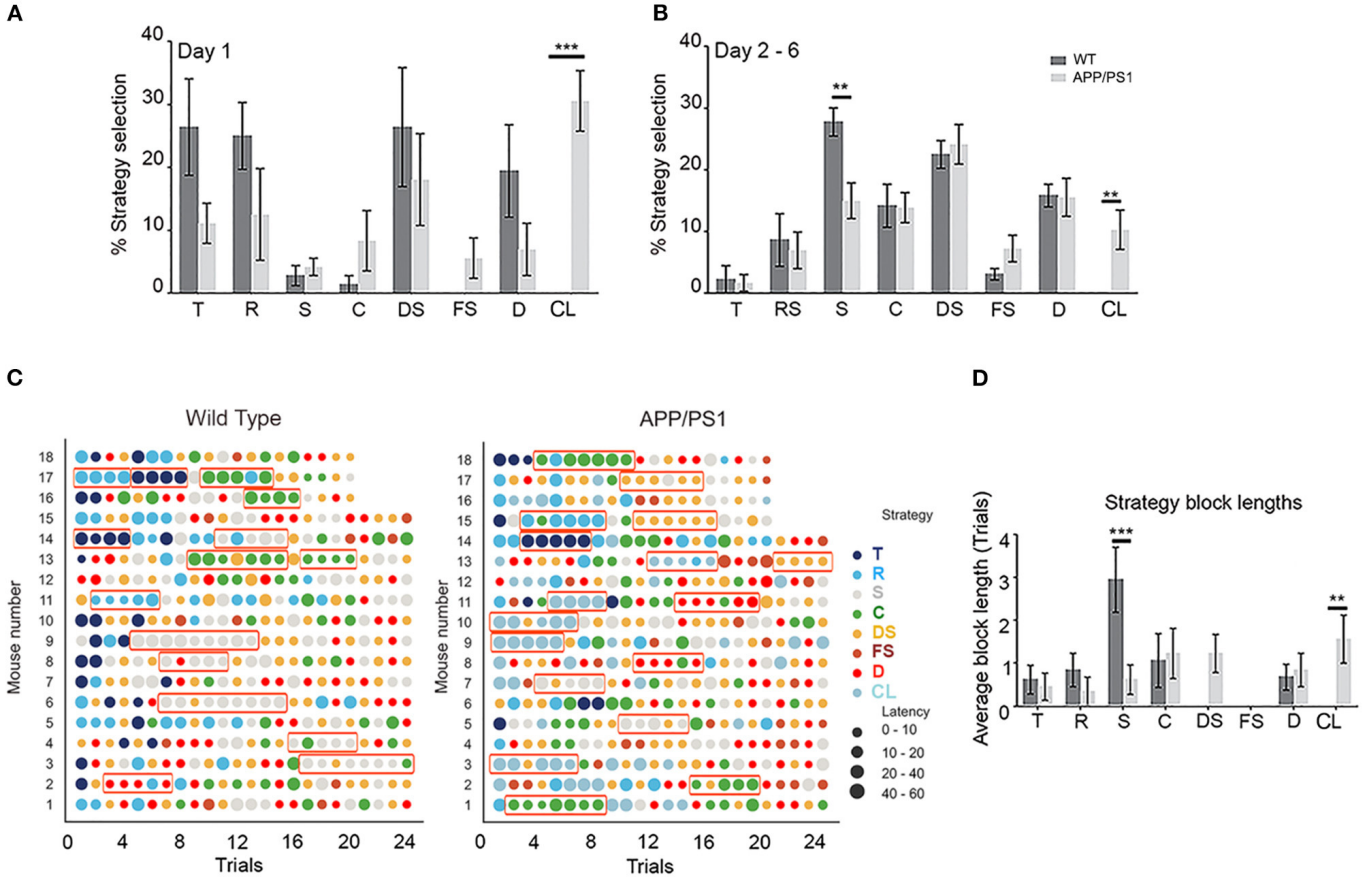
Percentage changes in search strategies between wild-type (WT) *vs*. APP/PS1 mice. The percentage changes in search strategies between WT *vs*. APP/PS1 during day 1 **(A)**, followed by days 2–6 **(B)**, of training are depicted. **(C)** A scatter plot of varying latency (size) *vs*. strategy (color) across all trials in individual animals. The greater the size of the circle, the more the latency. Strategy block length is defined as a sequence of at least three trials with the same strategy. Total block length is the sum of all blocks per strategy per mouse and is highlighted with rectangular orange icons. The last four trial's representations are missing from WT animal nos. 16, 17, and 18 and APP/PS1 mice nos. 15, 16, 17, and 18 due to the bad quality of the recorded videos, which thus could not be used for strategy analysis. **(D)** Average total block lengths for each search strategy during water maze learning. Data are presented as mean ± SEM; *n* = 18. **P = 0.0021, ***P = 0.0002.

The WT mice exhibited a consistent evolution in their search strategies over successive days of training (Figure 2B; Supplementary Figures 3A,B). They had three learning phases that made them more proficient at MWM learning. A detailed analysis of 18 individual learning curves revealed that most WT mice began with thigmotaxis or random swim, followed by local search strategies such as scanning and chaining that were predominant during days 3 and 4 of learning. Finally, on days 5 and 6, they opted for spatial search strategies such as directed search, focal search, and direct swim (Figure 2C; to the left). These shifts in strategy selection were explicit and distinct in WT mice. In contrast, the APP/PS1 mice exhibited disrupted transitions of strategies. The APP/PS1 mice began with an increased preference for circling over thigmotaxis or random swim, followed by an increased preference for directed search over local strategies, such as scanning and chaining, during days 2–4. Finally, on days 5 and 6, their preference swayed between scanning, directed search, and directed swim (Figure 2C; to the right). The search pattern utilized by APP/PS1 also indicated that they successfully used shortcuts by applying highly spatial and less challenging strategies, leading to the reduced deployment of search habits. This resulted in statistically significant differences in strategy implementation compared to WT mice [Figure 3A; day 1: two-way repeated-measures ANOVA: strategy: *F*_(7, 21)_ = 2.52, *P* = 0.0477; genotype effect: *F*_(1, 3)_ = 0.4357, *P* = 0.5563; interaction: *F*_(7, 21)_ = 10.94, *P* < 0.0001]. Tukey's multiple-comparisons test further revealed significant differences between WT and APP/PS1 mice with regard to preference for circling strategy (*P* = 0.0002). No statistically significant differences were noted in the preference for thigmotaxis between WT and APP/PS1 (*P* = 0.1562), unlike the 8-month-old APP/PS1 mice who exhibit significantly higher thigmotaxic swims than their age-matched controls during visible platform testing (Janus et al., 2015). From days 2 to 6, the trend in percentage changes with regard to strategy preferences for WT and APP/PS1 mice was even clearer [Figure 3B; days 2–6: two-way repeated-measures ANOVA: strategy: *F*_(7, 28)_ = 11.37, *P* < 0.0001; genotype effect: *F*_(1, 4)_ = 0.6759, *P* = 0.4572; interaction: *F*_(7, 28)_ = 6.563, *P* = 0.0001]. Tukey's multiple-comparisons test further revealed significant differences in preference for circling (*P* = 0.0035) and scanning (*P* = 0.0091) between WT and APP/PS1 mice. This data also indicated that WT mice utilized directed search and direct swim strategies beginning from day 1, and their percentage changes across days 2–6 of training were quite stable (Figures 2B,C, 3A). However, the APP/PS1 mice exhibited incremental percentage increases in directed search and direct swim strategies across days of training.

Circling was the more prominent non-spatial searching strategy in APP/PS1, along with thigmotaxis and random swim, which incrementally decreased over the days of training (Figures 2B, 3A,B). During circling, APP/PS1 mice made tight 360° loops after being introduced into the pool, which was maintained across trials. Circling traversed most of the pool area, but not within the range of distances that would result in efficient arrival at the platform. The notably higher number of unsuccessful trials during learning in APP/PS1 could be a result of this unpredictable circling strategy, leading to variations in latencies. Deployment of these strategies was identical to habit learning where there was repeated use of the same search strategy in at least three consecutive trials interrupted by one or two trials involving an alternative called strategy blocks (Figures 3C,D). Circling was repeated as the preferred search strategy in at least three consecutive trials interrupted by one or two trials involving an alternative in APP/PS1 mice. The average strategy block length across trials from day 1 to 6 [two-way repeated-measures ANOVA followed by Tukey's multiple-comparisons test: strategy: *F*_(7, 119)_ = 3.184, *P* = 0.0040; genotype effect: *F*_(1, 17)_ = 0.1175, *P* = 0.7360; interaction: *F*_(7, 119)_ = 3.984, *P* = 0.0006] further indicated statistically significant differences in both scanning (*P* = 0.0262) and circling (*P* = 0.0450) strategies between APP/PS1 and WT (Figures 3B,C). This data clearly indicates that circling is the preferred non-spatial strategy utilized by APP/PS1 mice over the scanning strategy opted for by WT mice.

### Evaluation of Reversal Learning in APP/PS1 Mice Using the MWM

**Figure 4:**
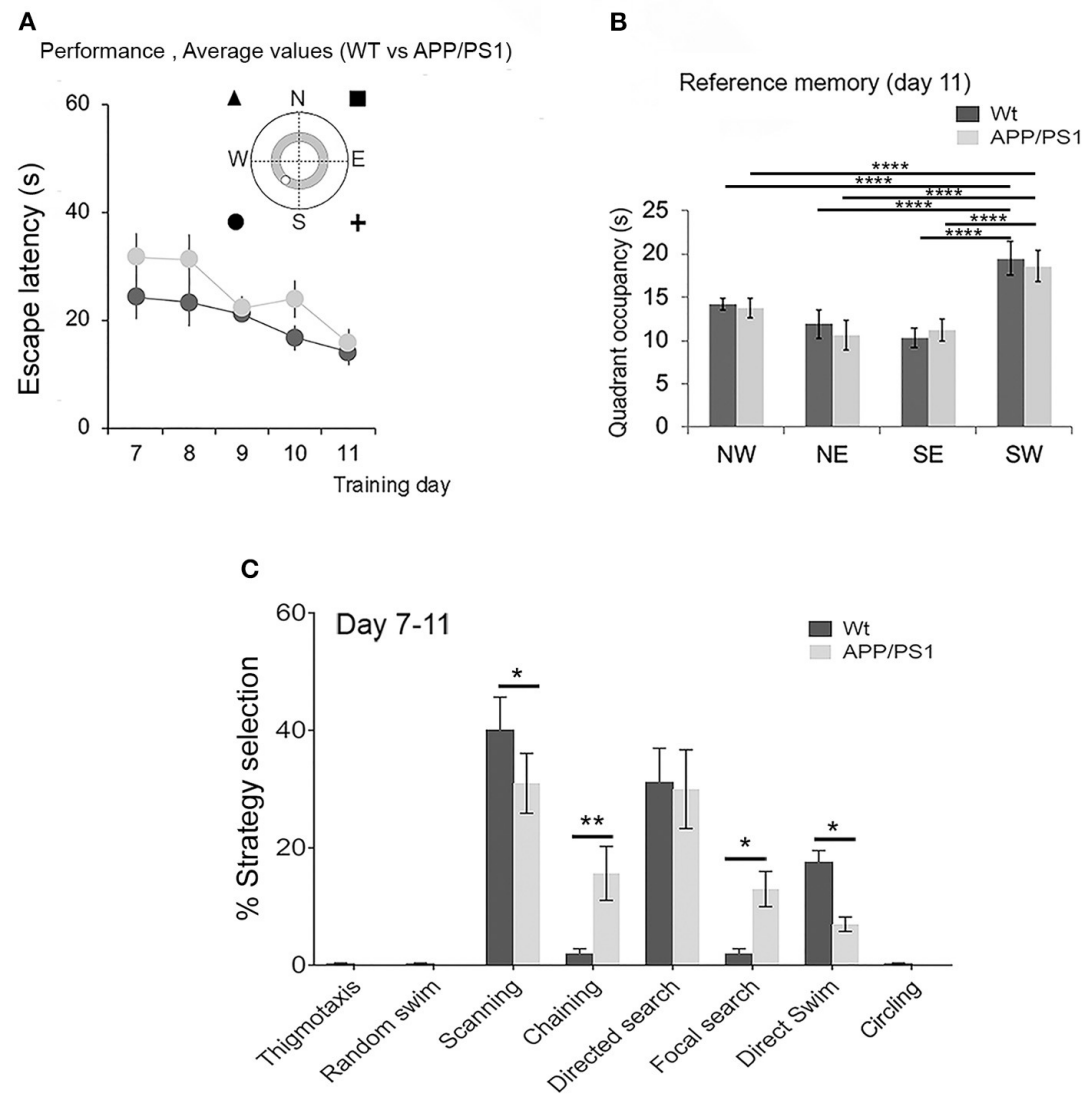
Evaluation of reversal learning in APP/PS1 mice using the Morris water maze. **(A)** Acquisition of reversal learning where the platform was placed in the opposite SW quadrant. Performance was evaluated as average escape latency between 2-month-old wild-type (WT; dark circle) and APP/PS1 (light circle) mice. **(B)** Reference memory was evaluated 2 h after the last trial on day 11. **(C)** Percentage changes in search strategies between WT *vs*. APP/PS1 during days 7–11 of reversal learning. Data are presented as mean ± SEM; *n* = 18. *P = 0.0332, **P = 0.0021, ****P < 0.0001.

We further challenged the APP/PS1 mice with reversal learning to assess their ability to reuse strategies that have already been established. Therefore, on day 7, after 6 days of acquisition learning, the platform was hidden in the opposite quadrant (SW), and the mice were trained to learn this new hidden platform position with unchanged extra-maze context and cues. The escape latencies declined in both groups over days of training from day 7 to day 11 [Figure 4A, right; two-way repeated-measures ANOVA: training effect: *F*_(4,68)_ = 10.86, *P* < 0.0001; genotype effect: *F*_(1,17)_ = 3.972, *P* = 0.0626; interaction: *F*_(4,68)_ = 1.228, *P* = 0.3074]. Tukey's multiple-comparisons test did not indicate any significant differences between the groups. After 5 days of reversal, both the APP/PS1 and the WT mice exhibited a tendency to prefer the SW target quadrant over other quadrants during the probe trial [Figure 4B; two-way ANOVA: *F*_(3, 136)_ = 29.2, *P* < 0.0001; genotype effect: *F*_(1, 136)_ = 0.0557, *P* = 0.1853; interaction: *F*_(3, 136)_ = 0.4437, *P* = 0.7222]. Tukey's multiple-comparisons test further revealed significant differences between the time spent in the target quadrant (SW) *vs*. the NE, SE, and NW quadrants in both WT and APP/PS1 mice. Deficits in their behavior became more apparent when we analyzed the navigational strategies that they opted for during reversal learning. The WT mice displayed a very sharp and selective search strategy which involved scanning, directed search, and direct swim predominantly. However, in APP/PS1, the choice of strategies swayed between scanning, chaining, directed search, focal search, and direct swim, leading to statistically significant differences in strategy deployment [Figure 4C; two-way repeated-measures ANOVA: strategy: *F*_(7, 28)_ = 20.59, *P* < 0.0001; genotype effect: *F*_(1, 4)_ = 0.0643, *P* = 0.8122; interaction: *F*_(7, 28)_ = 10.91, *P* < 0.0001]. Tukey's multiple-comparisons test revealed a significant difference in preference for scanning (*P* = 0.0326), chaining (*P* = 0.0027), focal search (*P* = 0.0123), and direct swim (*P* = 0.0180) between WT and APP/PS1 mice. Taken together, the APP/PS1 mice, unlike the WT mice, exhibited notably unpredictable trajectories to reach the platform.

## Discussion

In this study, we analyzed the average learning patterns of 2-month-old male APP/PS1 mice in the MWM task. This study is novel as it provides a detailed quantitative search strategy analysis which is crucial to understand the early stages of disease pathogenesis in APP/PS1 mice. Consistent with previous reports (Morris, 1984; Wolfer and Lipp, 2000; Garthe et al., 2009; Ruediger et al., 2012), detailed behavioral analysis revealed that WT mice utilized qualitatively different search strategies as they became more proficient at the MWM task. However, APP/PS1 mice exhibited an overt dependence on non-spatial strategies, especially circling, which led to distorted learning transitions. We did not observe any significant differences with regard to average learning patterns and strategy preferences between 2-month-old female WT and APP/PS1 mice (data not shown). Similar male-specific differences with regard to hippocampal metabolism (Agostini et al., 2020) and mGluR5 signaling (Abd-Elrahman et al., 2020) were recently demonstrated in this mouse model, further indicating that gender is an important modifier of AD progression.

Successful navigation to the hidden platform involves dynamic interactions between the hippocampal and striatal systems, enabling fluid transitions of navigational behavior. The increased dependence of APP/PS1 on less favorable strategies during learning might indicate deficits in feedback mechanisms in the striatal circuits mediating habit behavior (Graybiel and Grafton, 2015). The increased prevalence of circling may also be due to false recognition, impaired familiarity for the visual cues that guide the mice to the platform (Romberg et al., 2012), or an artifact of APP and PS1 overexpression influencing changes in gene expression, especially in the retina, at 2 months of age (Chintapaludi et al., 2020). The deficits were more apparent during the reversal learning trial (Figure 4) where previously adopted spatial search strategies or rules were successfully implemented faster by WT mice than APP/PS1 mice (Figure 4C). Therefore, the ability to efficiently adjust to reversal learning or behavioral flexibility also seemed compromised very early in APP/PS1 mice. However, with repeated trial and error, they eventually learned to find the hidden platform.

The repeated circling behavior in APP/PS1 mice was also reminiscent of topographical disorientation, which is common in human patients affected by mild cognitive impairment progressing to AD (Huang et al., 2002; Trivedi et al., 2006; Seo et al., 2007; Whitwell et al., 2007; Desikan et al., 2008). Furthermore, 41.9% of patients diagnosed with mild cognitive impairment have reported getting lost on a regular basis (Lim et al., 2010). This is associated with gray matter loss primarily in medial temporal regions such as the hippocampal complex, the amygdala, and the fusiform gyrus that affect orientation skills without causing severe and selective cognitive defects (Whitwell et al., 2007). In APP/PS1 mice, synaptic dysfunction and loss of synapses are documented in the cortex and hippocampus prior to pathophysiological changes (da Silva et al., 2016; Kommaddi et al., 2018). Since the APP/PS1 mice constitutively overexpress APP and PS1 genes, soluble oligomeric fractions of Aβ are present in their brain at 1–1.5 months of age (Ahmad et al., 2017). Elevated levels of toxic soluble oligomeric Aβ and synaptic deficits are also reported at 3.5 months of age in the cortex and hippocampus of APP/PS1 mice (Klein et al., 2001; Shemer et al., 2006; Hu et al., 2010; Ahmad et al., 2017). These deficits may underlie the mild behavioral impairments observed in this mouse model at a young age. Previous studies using APP/PS1 mice have not addressed these differences, owing to their focus on the endpoints of this complex learning paradigm (Table 2). Reference spatial memory deficits have been reported in 2- to 3-month-old APP/PS1 mice using cheeseboard maze (Pillay et al., 2008). However, the APP/PS1 mice in this study displayed a significantly decreased motivation to seek food compared to their WT counterparts, which could have been a confounding factor affecting exploratory behavior. Studying the exploratory strategies in such a scenario would provide insight into real cognitive deficits as well.

Amyloidogenic models are known to exhibit hyperactivity, which can potentially interfere with cognitive behavior readouts (Moechars et al., 1996; Dodart et al., 1999; Lalonde et al., 2003; Van Dam et al., 2003; Kobayashi and Chen, 2005). Therefore, we further evaluated the mice for hyperactivity by measuring the distance traveled and the average speed during MWM training (Supplementary Figures 2A,B) and did not find any significant differences between WT and APP/PS1 mice. Furthermore, open-field experiments report no hyperactivity in 6- to 18-month-old APP/PS1mice (Lalonde et al., 2004; Cao et al., 2007; Sood et al., 2007). Our results demonstrate that, even at 2 months of age, APP/PS1 display mild behavioral impairments in MWM. They further suggest that existing behavior assessment methodologies used to evaluate AD models must be systematically re-evaluated in light of our current understanding of AD progression and pathogenesis.

We recognize that our study has limitations. Firstly, it is limited to a single animal model of AD-like amyloidosis. Furthermore, we have not investigated a molecular mechanism that might explain the anomalous search strategies. Nevertheless, the findings in this study indicate the significance of early subtle readouts, which will be beneficial to tailor more informed treatment strategies in the future.

## Data Availability Statement

The original contributions presented in the study are included in the article/Supplementary Materials, further inquiries can be directed to the corresponding author/s.

## Ethics Statement

The animal study was reviewed and approved by Institutional Animal Ethics Committee of the Indian Institute of Science, Bangalore.

## Author Contributions

SK devised, carried out, analyzed all experiments, and wrote the manuscript.

## Conflict of Interest

The author declares that the research was conducted in the absence of any commercial or financial relationships that could be construed as a potential conflict of interest.

## Supplementary Material

Supplementary Figure 1 Reference spatial memory is intact in APP/PS1 at 24 h after the last Morris water maze learning trial: **(A)** mean acquisition latencies to reach the platform on six consecutive days (averaged of four trials per day) among wild-type (dark circle) and APP/PS1 (light circle) mice. **(B)** Reference memory evaluated at 24 h after the last trial on day 6. Data are presented as mean ± SEM; *n* = 7. ^****^*P* < 0.0001.

Supplementary Figure 2 Average Morris water maze training performance. **(A)** The average of swimming speed (m/s) and **(B)** the average distance traveled (m) over 6 days of training between wild-type (dark gray) and APP/PS1 (light gray) mice. Data are presented as mean ± SEM; *n* = 18.

Supplementary Figure 3 **(A,B)** Average prevalence of each strategy by trials across days of training: experiments with male wild-type (WT) and APP/PS1 mice were conducted on two separate days with two batches of animals. Each batch had nine WT and nine APP/PS1 male mice. The results of two replicate (batches) experiments are represented. Note the consistent circling strategy preference of APP/PS1 during water maze learning. Data are presented as mean ± SEM; *n* = 9.
