# Supplementary figures and images for: Unraveling Early Signs of Navigational Impairment in APPswe/PS1dE9 Mice Using Morris Water Maze
Source: Front Neurosci. 2020 Dec 15;14:568200. doi: 10.3389/fnins.2020.568200 (PMC7770143; doi:10.3389/fnins.2020.568200)

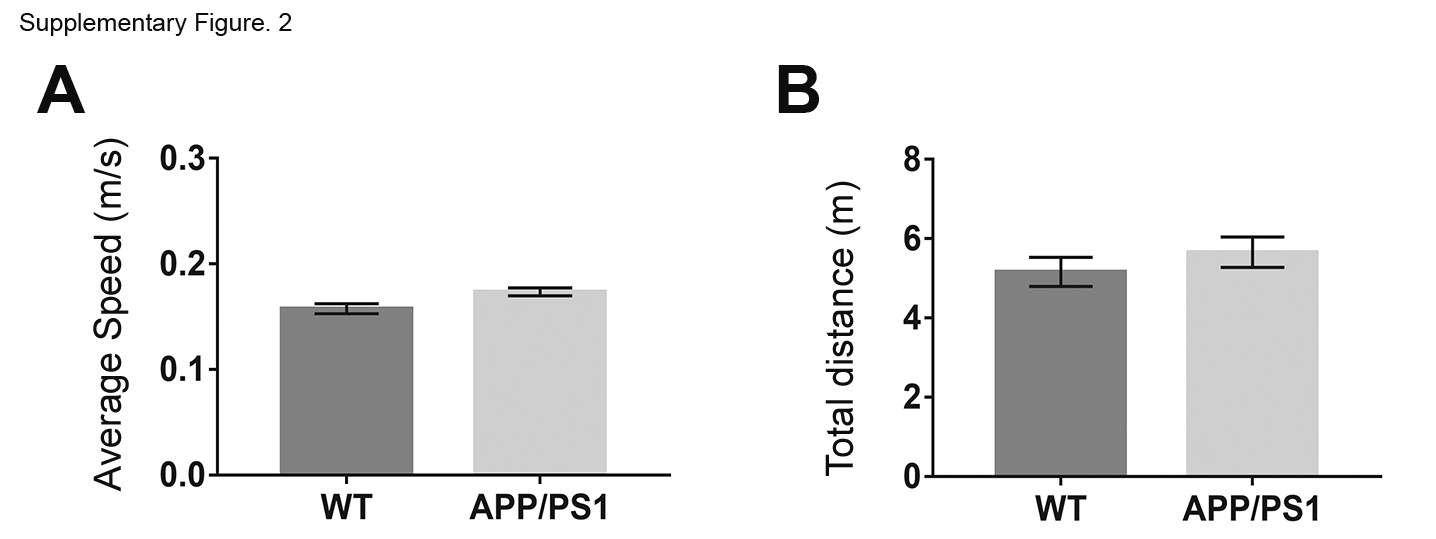

Supplement: Supplementary Figure 1 — Reference spatial memory is intact in APP/PS1 at 24 h after the last Morris water maze learning trial: (A) mean acquisition latencies to reach the platform on six consecutive days (averaged of four trials per day) among wild-type (dark circle) and APP/PS1 (light circle) mice. (B) Reference memory evaluated at 24 h after the last trial on day 6. Data are presented as mean ± SEM; n = 7. ****P < 0.0001. [file Data_Sheet_1.zip › Figure S2.TIF]

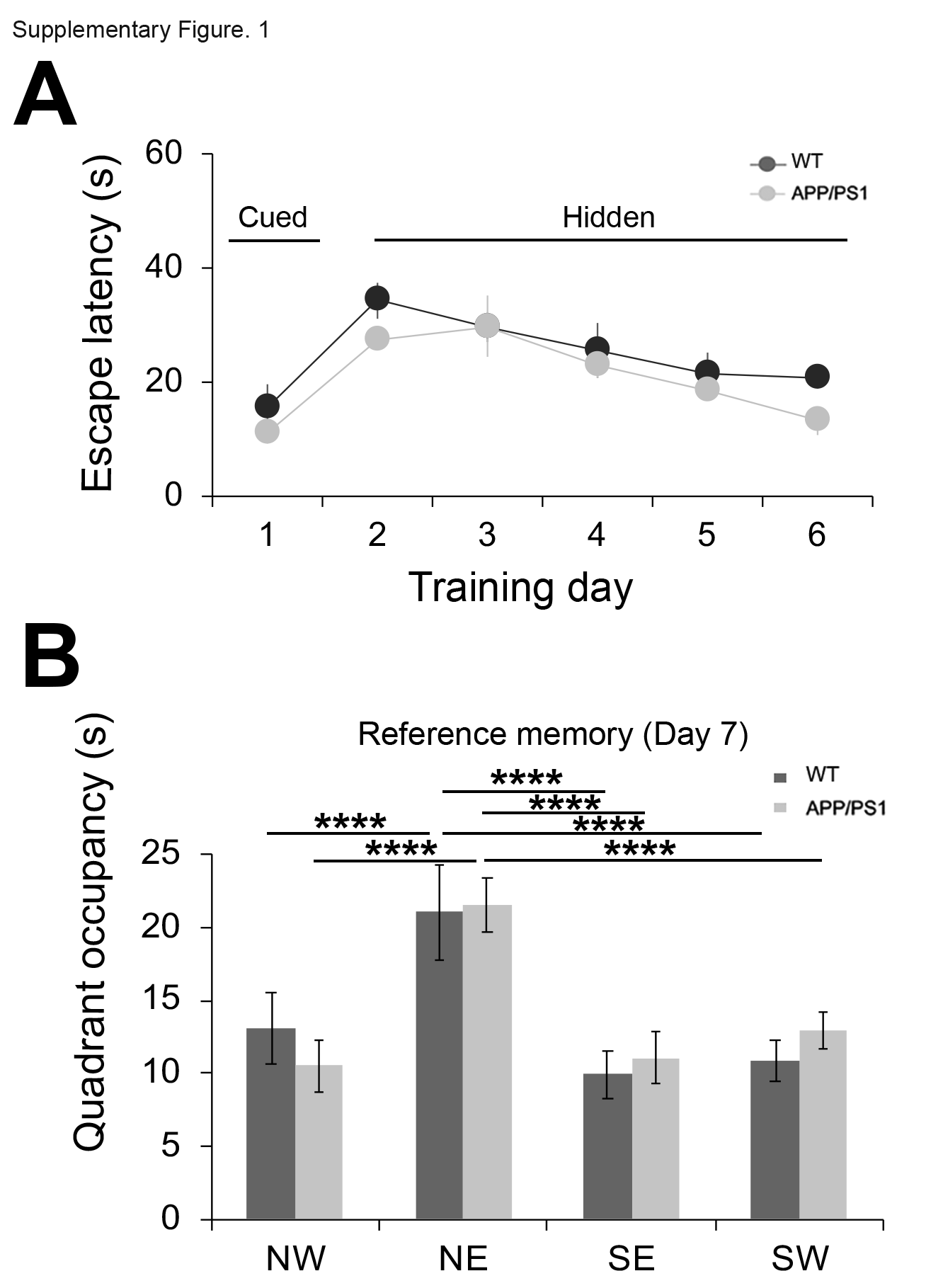

Supplement: Supplementary Figure 1 — Reference spatial memory is intact in APP/PS1 at 24 h after the last Morris water maze learning trial: (A) mean acquisition latencies to reach the platform on six consecutive days (averaged of four trials per day) among wild-type (dark circle) and APP/PS1 (light circle) mice. (B) Reference memory evaluated at 24 h after the last trial on day 6. Data are presented as mean ± SEM; n = 7. ****P < 0.0001. [file Data_Sheet_1.zip › Figure S1.TIF]
